# Assessing the Amount of Interdental Bone in Posterior Areas of the Mandible for Placing Orthodontic Mini-Implants

**Published:** 2013-05

**Authors:** Nasrin Esfahanizadeh, Damoun Shahraki, Hamidreza Daneshparvar, Ahmad Reza Talaei Pour, Mohammad Ali Saghiri, Ahmad Sheibaninia, Shadab Rashtak

**Affiliations:** 1 Associated Professor, Department of Periodontics, Islamic Azad University, Dental Branch, Member of Dental Implant Research Center, Tehran University of Medical Sciences, Tehran, Iran; 2 Post Graduate Student, Department of Orthodontics, Islamic Azad University, Dental Branch, Tehran, Iran; 3 Assistant Professor, Legal Medicine Research Center, Tehran, Iran; 4 Professor and Chairman, Department of Radiology, Islamic Azad University, Dental Branch, Tehran, Iran; 5 Assistant Professor, Department of Dental material, Member of Craniofacial Research Center, Islamic Azad University, Dental Branch and Kamal Asgar Research Center (KARC), Tehran, Iran; 6 Assistant Professor, Fellow of Orthosurgery, Department of Orthodontics, Azad University Dental School, Tehran, Iran; 7 Dentist, Dental Implant Research Center, Tehran University of Medical Scienses, Tehran, Iran

**Keywords:** Bone; Implant; Posterior Teeth

## Abstract

**Objective** **:** The aim of this study was to assess the amount of interdental bone in posterior areas of the mandible for placing orthodontic mini-implants to provide and control anchorage in orthodontic treatment.

**Materials and Methods** **:** The amount of interdental bone in areas between the second premolars and first molars, first and second molars on the right and left sides of the mandible were determined in fifty patients by RVG using periapical radiographs. The images were assessed using Cygnus Media Software to determine the mesio-distal width of the interdental bone, starting at the crest of the alveolar bone (2 mm below the CEJ) every one millimeter up to 12 mm from the CEJ. The actual amount of interdental bone and the effect of related factors were assessed using chi-square test at a 95% confidence interval.

**Results** **:** The minimum desired interdental bone width for placing mini-implants, 3 mm from the CEJ, between the second premolar and first molar and the first and second molars of the mandible on both sides were significantly different (p<0.01): 1.8 mm (31%) more apical in the area between the second premolar and the first molar. There was also a statistically significant difference between the areas under study on the right side (p<0.002), which was 2.2 mm (44%) more apical in the area of the second premolar and the first molar.

**Conclusion** **:** The most secure site for placing orthodontic mini-implants in the mandible is between the first and second molars at the height of 5.8 mm from the CEJ.

## Introduction

One of the necessities in orthodontic treatment is to provide and control anchorage. 

In order to provide secure and absolute anchorage in orthodontic treatment and circumvent the defects of older methods, current at tention is focused on using mini-implants [1]. 

**Fig 1:**
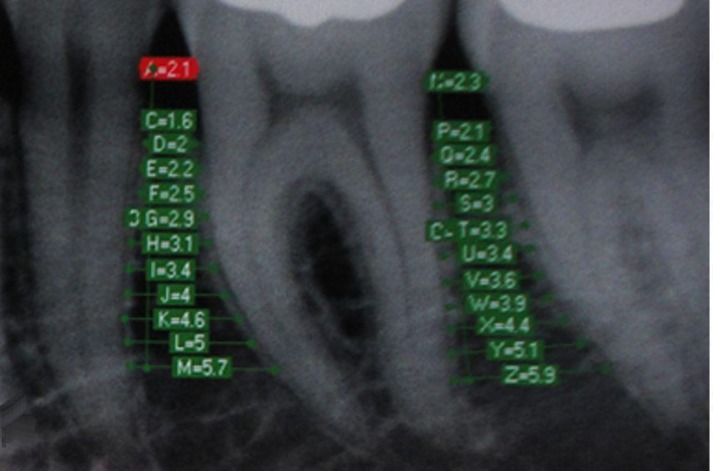
The interdental bone width measured with Cygnus Media

Orthodontic mini-implants are superior to other similar methods because of their unique characteristics such as easy placement, small size [2,3], durability, extraction without complicated surgical procedures, application of force immediately after placement, no need for toothless areas or patients’compliance and the low price [1-6]. 

Knowledge about the amount and condition of interdental bone for placement of orthodontic mini-implants is necessary and neglecting this results in destruction and damage to dental tissues, devitalization, and atrophy of the bone and tooth root [3,6,7]. Regarding the width of PDL (nearly 0.2 mm) [8], the diameter of mini-implants (1.2-2.0 mm) [6], and the fact that presence of 1 mm of bone around mini-implants will be sufficient for maintaining the health of periodontal tissues, tooth roots and durability of mini-implants within the bone, it is necessary to have at least 3-4 mm of interdental bone for placement of orthodontic mini-implants. 

Some studies [3,7] have shown that the long-cone parallel technique, using RVG in measuring the bone width, will result in more focused and sharper images, will have a precision of 0.1 mm and the dose of received radiation will be less than that in tomographic studies [9]. 

Various clinical and paraclinical studies have been performed on the placement site of mini-implants in interdental areas [2,3,6]. Differences in the results and lack of data in this regard in our community prompted us to assess the amount of interdental bone and related factors in posterior areas of the mandible for placement of orthodontic mini-implants in patients referred to the Radiology Department of Azad Dental School, Tehran Branch.

## MATERIALS AND METHODS

In 50 patients (23 males; mean age, 25.8 years and 27 females; mean age, 26.3 years) who were referred to the radiology department, supplementary examinations were carried out to match their condition with the requirements of the study, including lack of crestal bone atrophy, periapical lesions, caries, fillings, fractures, crowns or any other factors that obscured the CEJ. The study protocol was approved by the Ethics Committee and all the participants signed informed consent forms. Long-cone parallel technique was used to provide periapical radiographs in four dental areas (35 & 36, 36 & 37, 45 & 46, 46 & 47). The patients underwent radiography techniques using an RVG (Orix 65/10, Italy) set for obtaining the best image quality with the lowest exposure to the patients [9,14].

The imaging sensor (Cygnus Ray mps, Cygnus Technology) was held by a sensor holder (Endo-bite Sensor, Art. Nr. 2900, Kerr Total Care, USA ) in the patient’s mouth.

For each patient four images of the areas between the teeth 35 & 36, 36 & 37, 45 & 46, and 46 & 47 were obtained and saved in the patient’s file (Fig 1). Data were entered into the RVG computer and the resultant images were analyzed. In order to prevent technical errors, the process was entirely supervised by the Radiology Department and Dahlberg’s formula was used only in determining human errors during the study [6]. All the measurements were carried out by one clinician after one week. Twenty percent of the samples were selected randomly and the measurements were repeated by the same clinician. The images that lacked the required standards of radiography were excluded from the study and the imaging process was repeated after the patient’s consent [14].

To assess magnification and distortion of images in the study and in order to prevent measurement errors, the dry skull method was applied [9] using an acrylic stent with a mini-screw (1.6 mm in diameter and 6 mm in length) (Bone Screw, 16-JB-006, Jeil Medical Corporation, South Korea). The actual size of the mini-implant was measured again by digital calipers (Masel Digital Caliper) according to the manufacturer’s instructions.

In the next step, each image was measured by the Cygnus Media 3.0 Software according to standard methods 3,6; therefore, the process started from the alveolar crest (nearly 2 mm below the CEJ) in one millimeter intervals and was continued apically (between the PDL of the two adjacent teeth) to measure the amount of interdental bone up to 12 mm vertically from the CEJ. The amount of interdental bone in the sample was determined and its actual amount was estimated at a 95% confidence interval (CI). Then the relation between the type of teeth, gender, age and the amount of interdental bone was statistically judged using chi-square test. Paired t-test was applied to compare the interdental bone width between the two areas.

## Results

Of more than 600 patients, who underwent supplementary examinations, 50 had the required inclusion criteria and the others were excluded from the study. Table 1 shows that the mesiodistal width of the interdental bone of the first premolar and the second molar were not significantly different on the right and left sides (p<0.9) and the least amount of interdental bone suitable for insertion of mini-implants (3 mm in width) is located at a distance of 9 mm from the CEJ. 

The amount of interdental bone in the above-mentioned areas increases as the distance from the CEJ increases. In addition, Table 1 shows that the least mesiodistal width of interdental bone for insertion of mini-implants in the first and second molar areas of the right and left mandible are to be found at a distance of 6 and 7 mm from the CEJ, respectively. The amount of bone between the first and second molars on the right and left sides of the mandible at a distance of 2 mm from the CEJ increased apically, with no statistically significant differences between the right and left sides of the mandible (p= 0.9). Assessing the distribution of mesiodistal width of interdental bone (≤ 3) in the area between the second premolar and the first molar, as well as the first and second molars of the mandible, revealed no statistically significant differences among individuals with different genders and ages.

Table 2 shows that the vertical distance of interdental bone with the desired mesiodistal width (3 mm) in the second premolar and the first molar areas was located at a height of 7.6 (3.2) mm on the left side and 7.7 (3.3) mm on the right side from the CEJ. 

However, the desired mesiodistal width of the inter dental bone in the first and second molar

areas was found at a height of 5.8 (3.4) mm from the CEJ on the left side and 5.5 (3.1) mm from the CEJ on the right side, with statistically significant differences between the right and left sides of the mandible (p<0.01, p<0.9). 

In other words, the distances in the areas between the second premolar and the first molar on the left and right side were 1.8 mm and 2.2 mm, which were located 31% and 44% more apically, respectively.The distance of the desired area from the CEJ in the areas between the second premolar and the first molar, as well as the first and the second molar of the mandible on the left and right sides, exhibited no statistically significant differences (p<0.9).

The results of this study showed that the greatest amount of interdental bone is found between the first and the second molars of the mandible and this is a safe area for insertion of mini-implants. 

The most coronal proper mesiodistal width for insertion of mini-implants is in the area between the first and second molars of the mandible at a distance 5.8 (3.4) mm from the CEJ and between the first and second premolar it was more apical at a distance of 7.6 ± 3.2 mm from the CEJ. 

The results of this study showed that interdental bone increases in width at a distance of 2 mm from the CEJ when moving apically.

**Table 1:** The mesio-distal interdental bone width (between the mandibular second premolar and first molar, measured from the CEJ; Left), (between the mandibular first molar and second molar, measured from the CEJ; Right)

	**The mesio-distal interdental bone width between the mandibular first molar and second molar, measured from the CEJ**		**The mesio-distal interdental bone width between the mandibular second premolar and first molar, measured from the CEJ**	
**Cut Level from the CEJ Mean ± SD** **(mm)**	**Left Side**	**Right Side**	**Left Side**	**Right Side**
**2**	1.8**±**0.5	1.8**±**0.6	2.1**±**0.6	2.1**±**0.5
**3**	2.1**±**0.7	2.1**±**0.6	2.4**±**0.7	2.4**±**0.6
**4**	2.3**±**0.7	2.4**±**0.6	2.7**±**0.8	2.6**±**0.7
**5**	2.5**±**0.8	2.6**±**0.7	2.9**±**0.8	2.8**±**0.8
**6**	2.6**±**0.8	2.7**±**0.7	3.0**±**0.9	2.9**±**0.8
**7**	2.8**±**0.9	2.8**±**0.8	3.2**±**1.0	3.1**±**1.0
**8**	2.9**±**0.9	2.9**±**0.9	3.4**±**1.1	3.3**±**1.1
**9**	3.1**±**1.0	3.1**±**0.9	3.7**±**1.3	3.5**±**1.2
**10**	3.4**±**1.1	3.9**±**1.0	4.0**±**1.4	3.8**±**1.4
**11**	3.8**±**1.2	3.7**±**1.1	4.5**±**1.6	4.3**±**1.5
**12**	3.9**±**1.1	4.1**±**1.2	5.1**±**1.7	4.8**±**1.7

## Discussion

A number of studies have investigated the amount of internal bone with conflicting results [2,3,6,15]. The results of the present study are similar to those of a study by Hu et al. [3]. They showed that the distance between the roots increases from cervical to apical areas and is thickest at the area between the first and second molars of the mandible, and the safest site for insertion of mini-implants is found in the area between the first and second molars of the mandible at a distance of less than 5 mm from the cervical line. 

They reported that the proper site for insertion of mini-implants in the area between the second premolar and the first molar is located at a distance of 7 mm from the CEJ, consistent with the results of the present study. 

Meanwhile, Schnelle et al. [2] showed that the most coronal area with 3 mm of interdental bone is located in the area between the first and second molars of the mandible, which is consistent with the results of the present study. Their study revealed that most interdental areas, even after correction for magnification and measurement errors, have sufficient bone for mini-implants at a distance of more than one half of the length of the root apically, which is covered with alveolar mucosa. 

The present study confirms that between the second premolar and the first molars only four areas are safe for insertion of mini-implants, which is entirely in the apical third of the root, while six safe areas exist between the first and second molars, two of which are located in the middle third and others in the apical third of the root. Doldo et al. [15] showed that the safe site for insertion of mini-implants in the lower arch is between the second premolar and the first molar and between the first and second molars, which is located apically, consistent with the results of the present study. 

Hernandez et al. [16] reported that the area between the first and second molars of the mandible has the greatest interradicular mesiodistal width, which is consistent with the results of the present study. 

However, the amount of measured bone does not coincide with the results of the present study, which might be attributed to selecting different reference points for measurements (alveolar crest in Hernandez’s et al. [16] study and the CEJ in the current study). 

In addition, in the study carried out by Hernandez et al. [16] it is not clear whether the reference point for measurement of mesiodistal width of interradicular bone was root cementum or PDL space, while in the present study, the reference points for measuring the mesiodistal width of the interdental bone were the gaps between the PDLs of the two adjacent roots.

**Table 2:** Assessing the nearest cut level from the CEJ, showing 3 mm of mandibular interdental bone between different regions under study

**Cut level from the CEJ (mm)**	**Left Side**	**Right Side**
3 mm of Interdental Bone	7.6**±**3.2	7.7**±**3.3
Second Premolar and First Molar**±**SD		
First Molar and Second Molar**±**SD	5.8**±**3.4	5.5**±**3.1

Hernandez et al. [16] did not report presence or absence of atrophic changes in the crestal bone, while in the present study the patients with crestal bone atrophy were excluded. 

Poggio et al. [6] reported the highest amount of mesiodistal width of bone in the mandible between the first and second premolars at a height of 11 mm from the alveolar crest. 

They also showed that at a distance of 2 mm from the alveolar crest in the area between the second premolar and the first molar and the area between the first and second molars of the mandible, there are 3 and 3.2 mm of interradicular bone, respectively, while in the present study, the least amount of desired interdental bone (3 mm) was located at a distance of 7.7 and 5.8 mm, respectively, between the second premolar and the first molar and between the first and second molars of the mandible. The differences between the result of the present study and a study carried out by Poggio et al. [6] might be attributed to differences in the methods of imaging, i.e. CT versus periapical radiography. Generally it is believed that CT images do not provide precise measurements due to vague delineation of anatomic structures because of crowns, fillings and orthodontic instruments, which make diagnosis of reference points for measurements very hard. In CT images, there is no definite border between the alveolar bone and cementum and it is unnecessarily expensive and there is high radiation exposure [17,18]. 

Previous studies [19] have shown that periapical radiography (long-cone parallel technique) using a film holder is a simple, affordable and reliable method to assess the amount of interdental bone for insertion of mini-implants in the alveolar bone. One of the advantages of the present study was supplementary examinations to select subjects for this project that eliminated all the factors that might have caused disturbances in measuring interdental bone and determining reference points. Using long-cone parallel technique in periapical radiography by RVG [9,19] and using computer softwares and digital rulers [7] for measuring and magnifying the image to increase its sharpness and focus of reference points was another advantage of this study. Therefore, measuring errors decreased to 0.12 mm mesiodistally and 0.1 mm vertically. In addition, the amount of magnification of RVG was assessed and determined by placing a mini-implant (1.6 mm × 6 mm, Bone Screw 16-TB-006, Jeil Medical Corporation, South Korea) in an acrylic stent and imaging it showing a magnification of 0.34 mm vertically and 0.07 mm mesiodistally. The proper site for insertion of mini-implants is within the limits of the attached gingiva, but this study and other similar ones did not confirm the possibility of insertion of mini-implants in the areas between the teeth within the limits of attached gingiva [2,3] and in most areas, even after correcting for magnification and measurement errors, there was a sufficient amount of bone for insertion of a mini-implant in the middle and apical thirds of the root that is usually covered by alveolar mucosa. It should be pointed out that in the present study no measurement of the width of attached gingiva was performed and only papers on the normal width of the attached gingiva were reviewed [8,20,21,22, 23]. Maybe, if we had assessed the limits of the attached gingiva clinically, we had obtained more precise results in this regard.

## CONCLUSION

The results of the present study showed that the safest site for insertion of orthodontic mini-implants in the mandible is between the first and second molars at a distance of 5.8 mm from the CEJ.

## References

[1] Ludwig B, Baumgaertel S, Bohman SJ (2007). Chapter 2. Mini-implant in orthodontics.

[2] Schnelle MA, Beck FM, Jaynes RM, Huja SS (2004). A radiographic evaluation of the availability of bone for placement of mini screws. Angle Orthod..

[3] Hu KS, Kang MK, Kim TW, Kim KH, Kim HJ (2009). Relationships between dental roots and surrounding tissue for orthodontic mini screw installation. Angle Orthod..

[4] Carrillo R, Buschang PH, Opperman LA, Franco PF, Rossouw PE (2007). Segmental intrusion with mini-screw implant anchorage: a radiographic evaluation. Am J Orthod Dentofacial Orthop.

[5] Wilmes B, Su YY, Drescher D (2008). Insertion angle impact on primary stability of orthodontic mini-implants. Angle Orthod..

[6] Poggio PM, Incorvati C, Velo S, Carano A (2006). “Safe zones”: a guide for miniscrew positioning in the maxillary and mandibular arch. Angle Orthod..

[7] Lee YK, Kim JW, Baek SH, Kim TW, Chang YI (2010). Root and Bone Response to the Proximity of a Mini-Implant under Orthodontic Loading. Angle Orthod..

[8] Newman GM, Carranza AF (2007). Clinical periodontology.

[9] Talaei Pour AR, Mehralizadeh S, Mesgarzadeh A (2005). Comparison between conventional to-mography & radiovisiography methods for assessment of presurgical dental implants. J Dent Tehran Uni Med Sci..

[10] Kim JW, Baek SH, Kim TW, Chang YI (2008). Comparison of stability between cylindrical and conical type mini-implants. Angle Orthod..

[11] Park YC, Lee HA, Choi NC, Kim DH (2008). Open bite correction by intrution of posterior teeth with miniscrews. Angle Orthod..

[12] Baek SH, Kim BM, Kyung SH, Lim JK, Kim YH (2008). Success rate and risk factors associated with mini-implants reinstalled in the maxilla. Angle Orthod..

[13] Yamada K, Kuroda S, Deguchi T, Takano-Yamamoto T, Yamashiro T (2009). Distal movent of maxillary molars using miniscrew anchorage in the buccal inter radicular region. Angle Orthod..

[14] White SC, Pharaoh MJ (2009). Oral Radiology: Principles and Interpretation.

[15] Doldo T A, Costa A, Vessio V, Fazzari A (2007). Evaluation of bone thickness for inserting mini-screw in inter-radicular sites. Interna-tional Dentistry South Africa..

[16] Hernández LC, Montoto G, Puente Rodríguez M, Galbán L, Martínez V (2008). Bone map for a safe placement of miniscrews generated by computed tomography. Clin Oral Implants Res..

[17] Ekestubbe A, Thilander A, Grondahl K, Grondahl HG (1993). Absorbed doses from computed tomography for dental implant surgery: comparison with conventional tomography. Dentomaxillofac Radiol..

[18] Lecomber AR, Yoneyama Y, Lovelock DJ, Hosoi T, Adams AM (2001). Comparison of patient dose from imaging protocols for dental implant planning using conventional radiography and computed tomography. Dentomaxillofac Radiol..

[19] Burstein J, Mastin C, Le B (2008). Avoiding injury to the inferior alveolar nerve by routine use of intraoperative radiographs during im-plant placement. J Oral Implantol..

[20] Lang NP, Loe H (1972). The relationship between the width of keratinized gingival and gingival health. J Periodontol..

[21] Schroeder HE (1991). Dental attachment apparatus.

[22] Shaju Jacob P, Zade RM (2009). Width of attached gingiva in an Indian population: a descriptive study. Bangladesh J Med Sci..

[23] Ngan DC, Kharbanda OP, Geenty JP, Darendeliler MA (2003). Comparison of radiation levels from computed tomography and conventional dental radiographs. Aust Orthod J..

